# Advances in Development and Application of Influenza Vaccines

**DOI:** 10.3389/fimmu.2021.711997

**Published:** 2021-07-13

**Authors:** Jidang Chen, Jiehuang Wang, Jipei Zhang, Hinh Ly

**Affiliations:** ^1^ School of Life Science and Engineering, Foshan University, Foshan, China; ^2^ Department of Veterinary & Biomedical Sciences, University of Minnesota, Twin Cities, MN, United States

**Keywords:** influenza vaccines, viral vectored vaccines, seasonal influenza vaccine strains selection, universal influenza vaccines development, subunit vaccines

## Abstract

Influenza A virus is one of the most important zoonotic pathogens that can cause severe symptoms and has the potential to cause high number of deaths and great economic loss. Vaccination is still the best option to prevent influenza virus infection. Different types of influenza vaccines, including live attenuated virus vaccines, inactivated whole virus vaccines, virosome vaccines, split-virion vaccines and subunit vaccines have been developed. However, they have several limitations, such as the relatively high manufacturing cost and long production time, moderate efficacy of some of the vaccines in certain populations, and lack of cross-reactivity. These are some of the problems that need to be solved. Here, we summarized recent advances in the development and application of different types of influenza vaccines, including the recent development of viral vectored influenza vaccines. We also described the construction of other vaccines that are based on recombinant influenza viruses as viral vectors. Information provided in this review article might lead to the development of safe and highly effective novel influenza vaccines.

## Introduction

Influenza A viruses are enveloped RNA viruses with a viral genome (1) consisting of eight segmented negative-sense single-stranded RNAs encoding 10 core proteins known as Polymerase basic 2 (PB2), Polymerase basic 1 (PB1), Polymerase acidic (PA), Hemagglutinin (HA), Nucleoprotein (NP), Neuraminidase (NA), Matrix proteins (M1 and M2) and non-structural proteins (NS1 and NS2) and some other functional accessory proteins, such as Polymerase basic 1-F2 (PB1-F2), Polymerase basic 1 N40 (PB1N40), Polymerase acidic-X (PA-X), et al. (2–8). The polymerase tripartite complex consisting of PB1, PB2 and PA forms an RNA ribonucleoprotein (RNP) with the viral RNA and NP protein (9), which is responsible for the transcription and replication of the viral RNAs (10). HA, NA and M2 proteins are embedded in the outer envelope of the virus particle, in which HA and NA are readily exposed and therefore are two major immunogenic proteins. During the infection process, HA plays an important role in the viral entry step, such as attachment and penetration. HA is also the main antigenic glycoprotein on the surface of the influenza virus that can stimulate host neutralization antibody responses (11–14). NA is responsible for cleaving the terminal sialic acid residues present on cell surfaces, thus facilitating the release of progeny virions from the infected cells and playing an important role in the spread of influenza viruses (15). Based on the antigenic diversity of HA and NA, influenza viruses are classified into 18 HA subtypes (H1-H18) and 11 NA subtypes (N1-N11), respectively (16–18).

According to the World Health Organization estimates, about 3-5 million people have been infected with influenza globally that has resulted in approximately 290,000-650,000 fatalities due to severe respiratory disease-related complications (https://www.who.int/). However, influenza virus infection usually causes a common seasonal disease that is characterized by less severe clinical manifestations, such as fever, sore throat, muscle weakness, and a series of relatively mild respiratory symptoms (including cough and running nose). Most patients recover from these symptoms within a week; however, in some population subgroups, such as elderlies, children, pregnant women, and patients with immunosuppressive diseases, influenza virus infection can be quite serious that can lead to death (19–21).

In the 20th century, 4 pandemics of influenza virus infections have been registered, including 1918 H1N1 pandemic (also known as the Spanish flu), 1957-1958 H2N2 pandemic (Asian flu), 1968-1969 H3N2 pandemic (Hong Kong flu), and another H1N1 pandemic in 2009 (Mexican or swine flu) (22–25). The 1918 Spanish flu has been estimated to cause about 2 billion infections and 21 million deaths worldwide (22, 26). In 1957-1958, an outbreak of H2N2 subtype influenza virus infection originated in Asia and then spread worldwide to cause about 1-4 million deaths (23). Ten years later, another flu pandemic caused by the H3N2 subtype influenza virus originated in Hong Kong that resulted in 1-3 million deaths globally (24). The latest 2009 Mexican or swine flu pandemic was caused by the H1N1 subtype influenza virus that affected 214 countries, costing about 200,000 lives (25, 27). Besides human influenza viruses that mainly include H1N1 and H3N2 (24, 25, 28), some influenza viruses can also infect other animal species, including swine influenza H1N1, H3N2, and H1N2 (29, 30) and avian influenza viruses of the H5, H7, and H9 subtypes (31, 32). It is worth noting that among the aforementioned flu pandemics, several were caused by the trans-species transmission of avian-originated influenza viruses, such as the Spanish influenza H1N1, Asian influenza H2N2 (33), and Hong Kong influenza H3N2 viruses (34).

Currently, there are several therapeutics available to treat influenza virus infections. Three groups of anti-influenza viral agents can be classified into the viral M2 ion channel blockers and the neuraminidase inhibitors (NAIs). The first antivirals licensed for the treatment of influenza virus infection were amantadine and rimantadine, two small molecule inhibitors derived from the adamantane drug class. These two drugs inhibit viral replication by serving as inhibitors of the viral M2 ion channel by exerting electrostatic hindrance and preventing H^+^ flow through the M2 channel into the virion. Because the M2 ion channel was blocked, the delivery of the virus ribonucleoprotein into the cytoplasm was prevented (35, 36). Even though amantadine and rimantadine were the two earliest licensed anti-influenza drugs, influenza virus quickly evolves into virus variants with the drug resistant phenotype by acquiring mutations at amino acid positions 27, 30, 31, and 34 in the transmembrane domain of M2. The virus resistance strains could be observed in 30%-50% of patients after 2-3 days of drug treatment (37–39). Since the emergence of adamantane-resistant viruses, inhibitors targeting the neuraminidase of influenza viruses have been developed. Zanamivir, oseltamivir, and peramivir, derivatives of N-acetyl-neuraminic acid (2,3-dehydro-2-deoxy-N-acetylneuraminic acid) are Federal Drug Administration (FDA)-approved NAIs (40). These NAIs bind to the active site of NA, competitively inhibiting the binding of NA to its substrate N-acetyl-neuraminic acid. Due to this inhibition, NA can no longer cleave N-acetyl-neuraminic acid from host proteins, thereby preventing virion release from the cellular surface membrane. As expected, natural mutations have occurred in NA of influenza virus in patients who have been treated with these NAIs. Two mechanisms of resistance to NA inhibitors have been described. The first involves mutations within the NA enzyme catalytic site that disrupt its direct interaction with the drug. The second involves mutations in the HA that reduce affinity for its receptor, thus compensating for the effect of the drug on NA activity (41). The most frequently observed mutations, such as H275Y and N295S (N1 numbering) for influenza A viruses of N1 NA subtype; R292K and E119V (N2 numbering) for influenza A viruses of N2 NA subtype and R152 and D198 N for influenza B viruses (42), are the NA mutations that confer NA inhibitor resistance to reduce sialidase activity and/or stability. Besides two main anti-influenza therapeutics mentioned above, some novel antiviral inhibitors such as polymerase inhibitors (e.g., favipiravir and ribavirin), HA inhibitors (e.g. RO5464466, arbidol, MBX2329, and MBX2546), NP inhibitors (e.g., Naproxen and curcumin) and host targeting agents (e.g., DAS18) have also been discovered and could serve as additional therapeutic options against influenza virus infections.

Even though there are several therapeutics available for use as anti-influenza treatments, vaccination against influenza virus is still one of the most effective methods for preventing and controlling influenza virus spreading, which can significantly reduce the incidence of influenza virus infection and related medical complications (43). The commonly used influenza vaccines include inactivated influenza vaccine, split-virion influenza vaccine, subunit influenza vaccine, virosome influenza vaccine, live attenuated influenza vaccine, and recombinant viral vectored influenza vaccines (44). In this review, we discuss the advantages and disadvantages of different influenza vaccines, the development of viral vector influenza vaccines, and the construction of other virus vaccines using influenza viruses as vectors so as to provide important insights for developing effective and safe novel influenza vaccines.

## Different Types of Influenza Vaccines and Their Advantages and Disadvantages

Influenza vaccine is an important measure for the prevention and control of influenza virus infection. Currently, the commercially available influenza vaccines and experimental influenza vaccines mainly include inactivated whole virus vaccine, split-virion vaccine, subunit vaccine, virosome influenza vaccine, live attenuated vaccine and recombinant live viral vectored vaccine.

### Live Attenuated Influenza Vaccine

**Figure 1:**
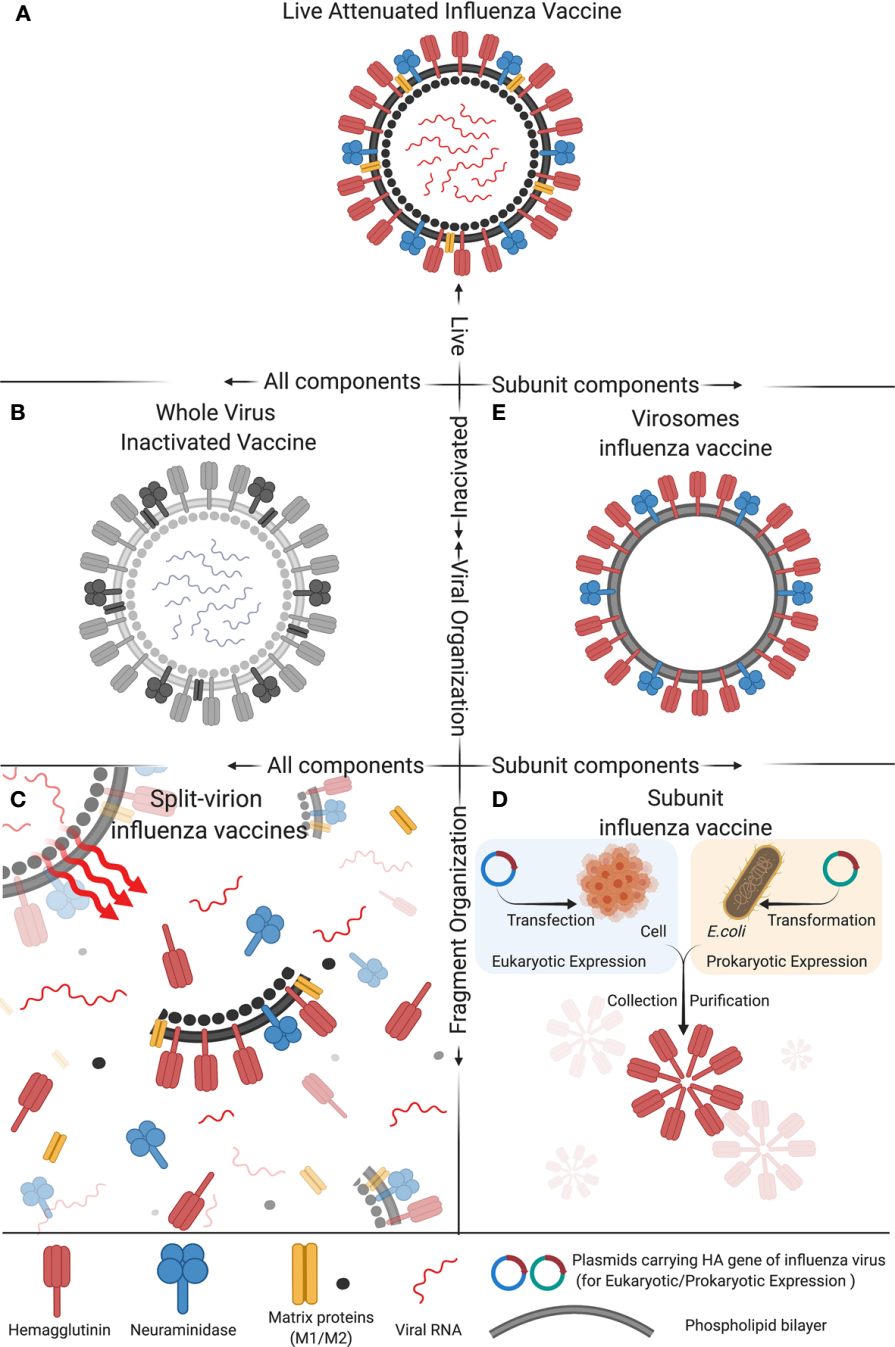
Composition of five types of commonly used influenza vaccines: **(A)** Live attenuated influenza vaccine, **(B)** Whole virus inactivated vaccine, **(C)** Spit-virion influenza vaccine, **(D)** Subunit influenza vaccine, and **(E)** Virosomes influenza vaccine. These five different compositions of influenza vaccine differ in activity, antigen components and structural organization, which impact the immunogenicity and protective efficacy of the vaccine. The figure was created with Biorender.com.

Live attenuated influenza vaccines (LAIV) are produced by the naturally occurring mutations in the influenza virus genome that generate less-virulent strains of influenza virus or by manually passaging a so-called parental influenza virus multiple times in virus-susceptible cells, embryonic chicken eggs or by other processes that make the virus less virulent, i.e., less likely to cause a significant disease in experimentally infected animals. The less-virulent strains of influenza virus are selected as the candidate vaccines for further testing in human volunteers before they can be considered by the FDA for approval for use as human influenza vaccines. This type of influenza vaccine strains has low virulence and pathogenicity but still allows the virus to infect and proliferate within a relatively short period of time after vaccination (**Figure 1A**) (45, 46). LAIV mainly includes vaccines for influenza A(H1N1), A(H3N2) subtype, and Victoria strains of influenza B virus. According to the data published to date, LAIV’s intranasal inoculation could induce mucosal immunity, humoral immunity, and cellular immunity. In particular, the cellular immune responses induced by LAIV, such as high cross-reactive CD8+ T-cell response and IFN-γ+ T cells response, in infants and children of school ages were better than in adults (47–51). With the advancement of reverse genetics techniques, i.e., molecular methods used in the laboratory setting to engineer viruses from circular pieces of DNA called plasmids that encode the entire genome of a virus, manual deletion or replacing the virulence genes of viruses are used in many studies to attenuate the virulence of viruses or to engineer viruses to carry one or more desired foreign genes (52–55). Using these modern techniques, trivalent and quadrivalent LAIVs can be generated to express three or four influenza viral HA genes, respectively. Quadrivalent LAIVs have been shown to provide effective protective effects for children of 2-17 years old in the USA (47). In China, the first trivalent LAIV (Changchun Bcht Biotechnology Co, China) was approved for marketing in 2020. However, considering that LAIV can still replicate in the vaccinated person, and therefore can potentially evolve its genome into a parental-like virus through a process called genetic reversion that may allow it to spread in the human population, LAIVs may present a potential risk to public health. Therefore, more studies are needed to investigate the safety of such LAIVs before extensive use (56, 57).

### Whole Virus Inactivated Vaccine

In the 1940s, the research group led by Thomas Francis, Jr., MD and Jonas Salk, MD at the University of Michigan developed the first influenza vaccine that was based on an inactivated influenza A virus (**Figure 1B**) and was licensed for military used in 1945. Their vaccine uses fertilized chicken eggs in a method that is still used to date to produce most influenza vaccines. This method involves cultivating the virus in chicken embryo (or eukaryotic cells) to allow virus proliferation, after which the virus is inactivated by heating or treating it with chemical reagents (mainly formalin) in order to attenuate the virus’s ability to infect cells and to proliferate. Adjuvants are then added to enhance the immunogenicity of the final vaccine formulation (58). The inactivated influenza viruses contained in the whole virus inactivated vaccine formulation are deprived of virulence and transmissibility, while the complete antigenicity provided by the viral surface protein antigens, such as HA and NA, is preserved. Therefore, inactivated whole influenza virus vaccine has the advantages of having a high safety profile, maintaining complete viral antigenic components with high immunogenicity, providing long duration of immunity, and having no risk of reversion to virulence (59, 60). Aforementioned, the advancement of reverse genetics approaches have revolutionized the process of preparing an inactivated whole influenza virus vaccine that consists of recombinant influenza viruses with known virulent genes deleted or manipulated in such a way to inactivate the viruses for use as whole inactivated vaccines. Besides human use, inactivated whole influenza virus vaccine is also used as an important tool in preventing avian influenza virus (AIV) infection in poultry industry (61).

Influenza vaccines for preventing avian influenza in China are mainly inactivated whole influenza virus vaccines. The six products of inactivated avian influenza vaccines that have been approved by the Ministry of Agriculture and Rural Affairs of the People`s Republic of China in 2018 (http://www.moa.gov.cn/gk/) are mainly targeting the H5 and H7 subtypes of AIV. These six vaccines include reassortant Avian Influenza Virus (H5+H7) Trivalent Vaccine and Inactivated (H5N2 Strain rSD57+Strain rFJ56, H7N9 Strain rGD76). These vaccine strains use the H5N2 subtype influenza viral strain (D7) as a viral genome backbone (provided six inner viral genes) and the HA and NA genes from the H5N1, N5N6, and H7N9 mutant virus strains, respectively. These recombinant vaccine strains are generated by using reverse genetics techniques, targeting several viruses that are mainly spreading in poultry farms in China, including H5 subtype viruses that carry the HA gene of clade2.3.2.1d and clade2.3.4.4d, as well as H7N9 virus, which produces high immunological effects in domestic fowls (62, 63). Similarly, the newly licensed recombinant vaccine that consists of inactivated reassortant Avian Influenza Virus (H5+H7) Trivalent Vaccine (H5N1 Strain Re-11+Strain Re-12 and H7N9 Strain H7-Re3), developed by the Harbin Veterinary Research Institute, Chinese Academy of Agricultural Sciences, was also generated through reverse genetics techniques (64). It is noteworthy that these AIV inactivated vaccines not only can prevent AIV infections in poultry species but also are of great benefit to human public health. The H7N9 subtype AIV caused hundreds of human case fatalities that began in 2013 and was therefore considered a major public health threat in China. However, after the introduction of the H5/H7 bivalent inactivated influenza vaccine into the poultry industry in September 2017 to control H7N9 influenza virus infections, the prevalence of H7N9 virus in poultry was drastically reduced (62, 65), and vaccination of chickens with this vaccine was also quite effective in preventing human infections with H7N9 virus as demonstrated by the fact that there were only 4 human H7N9 cases reported in the following two years after the H5/H7 bivalent vaccine was widely used with a 99.5% reduction compared to the number of human H7N9 cases documented between October 1, 2016 and September 30, 2017 (66, 67).

Inactivated whole influenza virus vaccines can induce humoral immunity *in vivo* and produce high-titer specific IgG antibodies. However, the cross-immune protective effects of such vaccines for viruses of different genotypes or subtypes are suboptimal (68). In addition, several other disadvantages, including relatively low rate of antibody production, relatively low effects of inducing cellular immune responses, and more immunizing doses that can induce excessive stresses to the vaccinated animals, have also been reported (69). It has also been noted that inactivated whole human influenza virus vaccines can induce high fever in children, and thus such vaccines are not recommended for those younger than 12 years old (70). With the advancements of vaccine production processes, the production of whole human influenza virus vaccines has been gradually abandoned. In contrast, the development and application of split-virion vaccines and subunit vaccines that can provide comparable and beneficial immunity effects and have a higher safety profile have advanced (57, 71, 72).

### Split-Virion Influenza Vaccines

Split-virion influenza vaccines are prepared based on the inactivated whole influenza vaccines. Appropriate splitting agents and conditions are selected to disrupt the viral envelope and split open the virion particles (**Figure 1C**). The splitting agents and the nucleic acids and large molecular weight proteins of the virus are discarded, while the active antigenic components (HA and NA) and part of M and NP proteins are preserved. Such a vaccine formulation can concentrate and increase the levels of active antigenic proteins in a given volume of the vaccine, which can stimulate maximum antibody production effects while greatly reduces the unnecessary side effects potentially caused by other components of the virion particle. Therefore, split influenza vaccines are safer than inactivated whole influenza vaccines (73, 74). Currently, the vaccines of this type that have been approved for marketing in China include the trivalent inactivated influenza vaccine (IIV3), and the quadrivalent inactivated influenza vaccine (IIV4) (44). Split influenza vaccines are the major component of IIV3, while all the IIV4 are split influenza vaccines (https://www.nifdc.org.cn/nifdc/fwzn/ppjpqf/index.html). Previous studies have shown satisfactory protective effects against influenza infection in children older than 6 months of age who received the split-virion immunization, with the statistics of the data in 2011-2012 vaccinations showing that the protective effects of IIV3 for children with the ages of 36-59 months and 6-35 months were 58.2% and 49.6, respectively (75, 76). In addition, other studies have also demonstrated that the immunogenicity of IIV4 for influenza B virus is higher than IIV3 (77, 78). The IIV4 mainly include vaccines for influenza A(H3N2), A(H1N1) subtype, and Victoria and Yamagata strains of influenza B virus. The positive seroconversion rate of hemagglutination inhibition (HI) as a measure of the presence of antibodies in the serum of the vaccinated individual that can neutralize influenza virus, average increase rate in Geometric Mean Titers (GMT) of HI, and serum antibody protection rate of the vaccines after immunization all meet the expectations, suggesting that the vaccines have high immunogenicity (73, 74, 79, 80).

### Subunit Influenza Vaccine

The traditional subunit vaccines are prepared based on the split-virion vaccines, for which the HA and NA antigenic proteins are further extracted, purified, and concentrated. Then, corresponding adjuvants are added to prepare the final subunit vaccine formulations (**Figure 1D**) (81). Alternatively, with the advancement of genetic engineering techniques, molecular cloning techniques have been applied to construct subunit influenza vaccines. In brief, the genes of major antigenic proteins of influenza viruses, such as HA and NA, are cloned into the protein expression plasmids through a process that involves restriction enzyme digestion and ligation (82). The engineered plasmids are transferred into either prokaryotic or eukaryotic cells to produce the HA or NA protein antigen, which is concentrated and purified, and corresponding adjuvants are then added to obtain the final subunit vaccines (**Figure 1E**) (83, 84). The commonly used IIV3 vaccines include some vaccines prepared by this method. For instance, the Influvac^®^ (Solvay Pharmaceuticals BV, Weesp, The Netherlands) and Agrippal^®^ (Chiron SRL, now Novartis Vaccines, Viaflorentina, Italy) vaccines have shown good immunogenicity, safety, and tolerability in healthy children, adults, and elderlies. As pregnant women are highly susceptible to influenza virus infection, trivalent influenza subunit vaccines Agrippal S1 and Grippol Plus are specifically designed to prevent influenza in pregnant women, which meet the Committee for Proprietary Medicinal Products (CPMP) standards and have been shown to provide high-titer antibodies for pregnant women and immune-protective effects for infants for as long as 6 months (85, 86). Besides human use, subunit influenza vaccines for swine and poultry have also been widely studied. Hernandez and colleagues (87) used the baculovirus vector system to express the swine influenza virus (SIV) HA protein combined with swine IgG2a Fc, which could provide effective anti-H1N1 SIV effects and high-titer serum antibodies for pigs and reduced pulmonary damages. Additionally, Song and colleagues (88) evaluated the immunogenicity of recombinant HA1-2 subunit vaccines for avian source H7N9 influenza and found that the inactivated vaccine candidates with adjuvants could induce effective HA1-2 specific humoral immune responses and cell-mediated immunity responses in mice. It is noteworthy that while subunit influenza vaccines are safe, they have several disadvantages, including relatively low immunogenicity, high vaccine doses needed, and high manufacturing costs, all of which need to be further improved.

### Virosomes Influenza Vaccines

Virosomes are reconstituted influenza virus envelopes consisting of HA, NA and viral phospholipids (**Figure 1E**). Their particulate structure enables virosomes to retain viral membrane fusion and cell-binding capabilities, which can increase their immunogenicity compared to subunit and split-virion vaccines (89). Virosomes can deliver encapsulated cargo into the cytosol of the antigen-presenting cells (APCs) and subsequently induce cytotoxic T lymphocyte (CTL) responses (90). However, due to a lack of adjuvants, virosomes are inefficient in activating APCs and thus in triggering cross-presentation, which limits the induction of CTL immunity. Therefore, adding suitable adjuvants can overcome this short coming (91). Dong and colleagues (91) have developed a novel influenza vaccine formulation consisting of virosomes with the Toll-like receptor 4 (TLR4) ligand monophosphoryl lipid A (MPLA) and the metal-ion-chelating lipid DOGS-NTA-Ni incorporated into the membrane. *In vitro*, virosomes with incorporated MPLA can induce stronger activation of APCs than unadjuvanted virosomes and immunization of mice with MPLA-adjuvanted virosomes with attached viral NP can result in priming of the NP-specific CTLs. Virosomes have also been used as delivery systems for short peptide antigens. Soema and colleagues (92) constructed virosomes with a conserved human HLA-A2.1 influenza T-cell epitope M158–66. The immunogenicity and protective effects of these peptide-loaded virosomes were assessed in HLA-A2 transgenic mice. Delivery properties of the virosomes were studied in mice and in dendritic cell cultures. Immunization of HLA-A2.1 transgenic C57BL/6 mice with peptide-loaded virosomes in the presence of the adjuvant CpG-ODN 1826 increased the number of peptide-specific CTLs. Vaccination with adjuvanted peptide-loaded virosomes reduced weight loss of mice after heterologous influenza virus challenge.

Since the virosomes influenza vaccine was first proposed in the 1970s (93), after more than 20 years of development, influenza virosome vaccines have gathered sufficient clinical experimental data and have resulted in two products Epaxal^®^ and Inflexal^®^ that were produced in 1994 and 1997 by the Swiss Serum and Vaccine Institute, Bern, Switzerland (94, 95). Among them, Inflexal V is an adjuvanted influenza vaccine for all age groups and shows good immunogenicity in both healthy and immunocompromised elderlies, adults and children (96). It can induce B cell responses and produce viral antigen-specific antibodies. However, a clinical study failed to demonstrate the induction of CD8+ T cells against two peptides (CTL target antigens) encapsulated into virosomes. Whether this shortcoming indicates a limitation of the virosome technology or merely reflects suboptimal target antigen selection remains unclear (97). Similar to the inactivated whole influenza vaccine, split-virion influenza vaccines, subunit influenza vaccine and virosomes influenza vaccines mainly induce B cell-induced humoral immunity. Future studies are needed to develop influenza vaccines that can induce both cellular and humoral immunity, such as the aforementioned live attenuated influenza vaccine and the live viral vectored vaccines described below.

## Viral Vectored Influenza Vaccines

Live viral vectored influenza vaccine refers to the use of genetic engineering technology to incorporate the antigenic protein(s) of influenza virus into the genome of a different type of virus, which allows continuous proliferation of this recombinant virus in the vaccinated hosts in order to ensure the high level of expression of the antigenic protein(s) of influenza virus. This consequently induces specific anti-influenza immune effects and/or non-specific innate immune effects in the vaccinated individuals (12, 98–100). The commonly used viruses (or viral vectors) to introduce influenza protein antigens into vaccinated host include but are not necessarily limited to adenovirus vectors, arenavirus vectors, Newcastle disease virus vectors, baculovirus vectors, and herpesvirus vectors. Similar to the aforementioned live attenuated influenza vaccines (LAIV), the advantages of live viral vectored vaccines include their ability to induce strong humoral and cellular adaptive immune responses against the immunogenic proteins as well as its ability to usurp the host cellular machinery for the purposes of expressing the antigenic proteins of choice. Consequently, these features can simultaneously induce humoral and cellular immune responses, produce antibodies within a relatively short period of time, maintain long immune duration, and therefore can exert high immune effects (57, 101, 102). In addition, live viral vectored vaccines could also be used to further construct polyvalent vaccines or multi-component vaccines, thus reducing the time of vaccine product development as well as simplifying vaccination and improving the cross-protective effects among viral strains of different genotypes (103, 104). Due to the advantages mentioned above, live vectored influenza vaccines have good prospects for further development. As such, they have gradually become a research hotspot for the production of many novel vaccines, including influenza vaccines. We will describe the different types of virus vectors used in vaccine development and their advantages and disadvantages below.

### Adenovirus Vectored Influenza Vaccines

**Figure 2:**
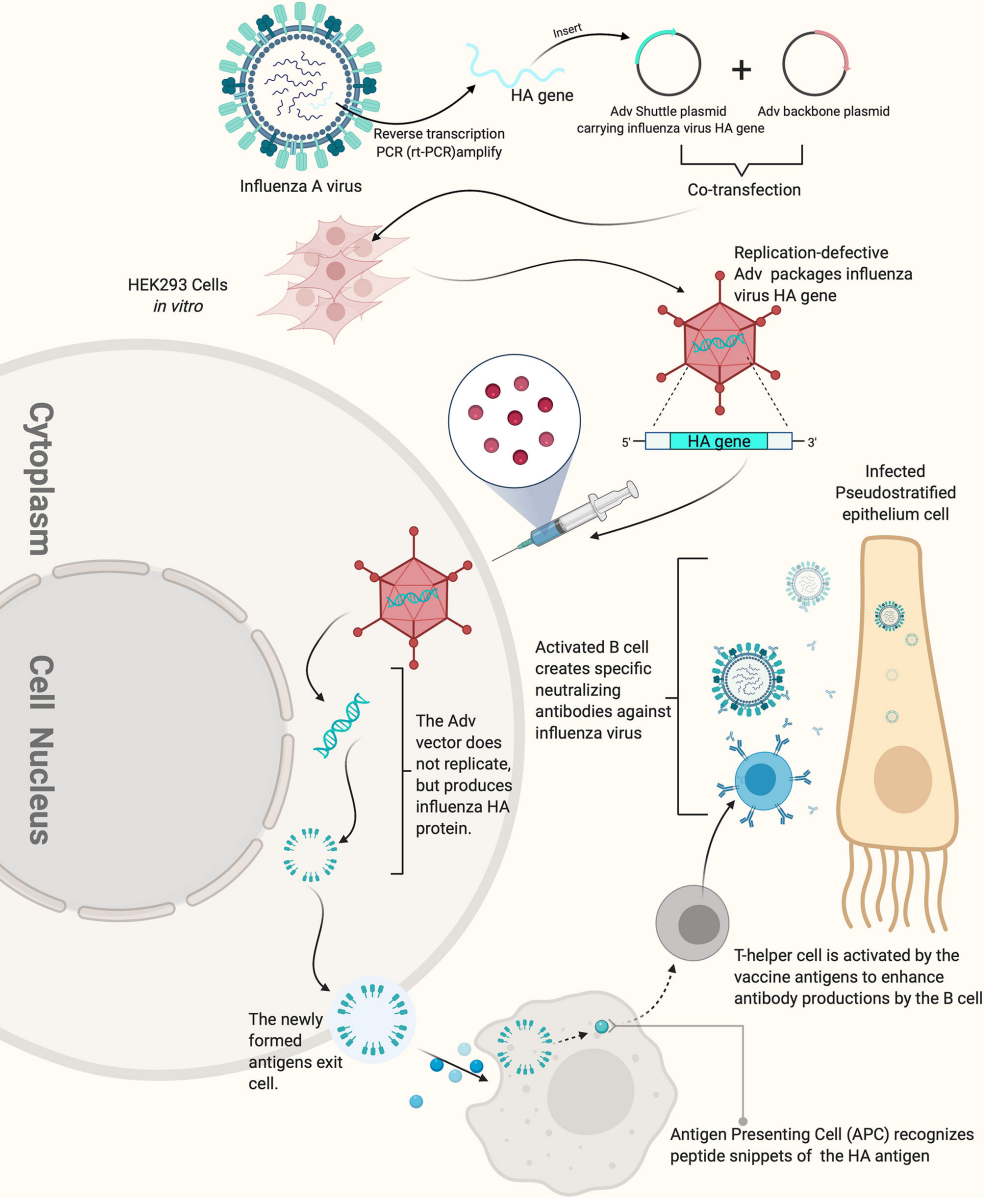
How replication-defective adenovirus vector influenza vaccine works. The replication defective adenovirus vector carrying the HA gene of influenza virus can induce the production of high-titer HI antibody. The figure was created with Biorender.com.

**Figure 3:**
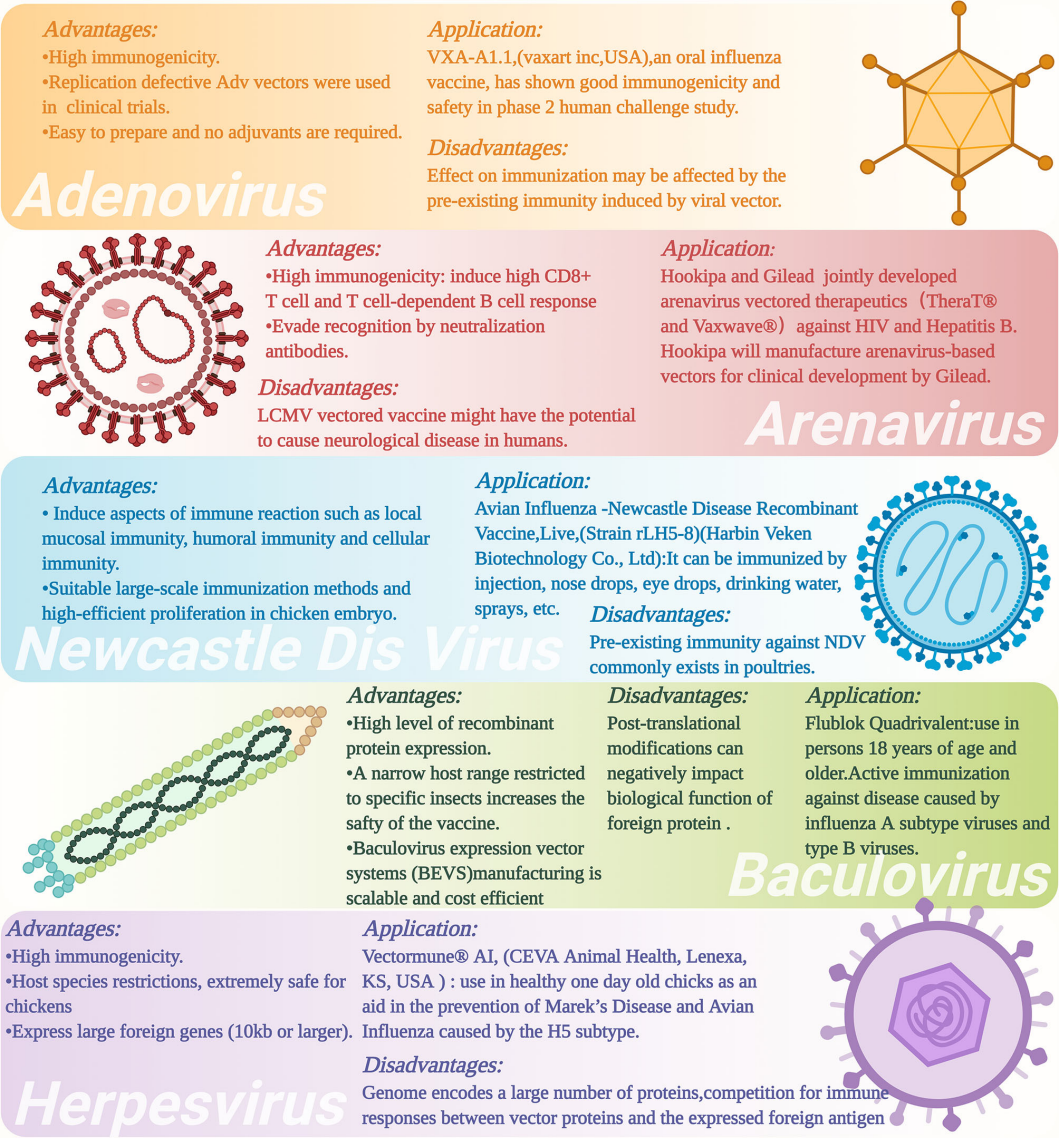
Advantages and disadvantages as well as applications of different types of viral vectors and viral vectored vaccines. The figure was created with Biorender (Biorender.com).

Adenovirus vectored vaccines use adenovirus as the vector to express the transgenes of protective antigenic proteins of the pathogens of interest by combining it into the adenovirus genome (**Figure 2**) (101). Adenovirus vectored vaccines have several advantages, including high expression levels of the transgene of the antigenic protein and the ability to simultaneously induce humoral and cellular immune responses (**Figure 3**). They are also safe, easy to prepare and do not require adjuvants. Currently, they are widely developed as prophylactic vaccines (105–107).

Adenovirus vectored vaccine strategy has been used in an attempt to develop universal influenza vaccines. Because influenza viruses include many genotypes (or subtypes) and can evolve relatively quickly through the processes of natural genetic mutations and/or genome reassortment known as antigenic drift and antigenetic shift, respectively (108), it can present a real challenge to produce a vaccine that can provide protection for an individual against different influenza viruses at the same time. Developing universal influenza vaccines is an important method for addressing this important issue. Toward this end, adenovirus vectored influenza vaccines have been constructed to express the conserved antigenic regions of influenza viral proteins, such as the stem region of HA2, chimeric HA, M2e, NP, and their T/B cell epitopes, as these major protein antigens have shown high immunogenicity and immune protective effects for various subtypes of influenza viruses (57, 109, 110). For example, Tutykhina and colleagues (109) constructed a recombinant adenovirus vector carrying the conserved M2 and NP antigenic epitopes of influenza virus (Ad5-tet-M2NP), which could induce the production of high-level CD8+ and CD4+ T cells, suggesting vaccination with this recombinant viral vector could induce potent T cell-mediated immune responses against the influenza virus. Mice vaccinated with this live adenovirus vectored vaccine could produce a high level of effector memory CD44+CD62- T cells 8 months later, and showed effective long-term protective effects of the vaccine against five types of influenza viruses (H9N2 (A/Swine/Hong Kong/9A-1/98), H1N1 (A/USSR/90/77), H5N2 (A/Duck(Mallard)/Pennsylvania/10218/84), H2N3 (A/BlackDuck/NewJersey/1580/78), and H3N2 (A/Aichi/2/68)). Hassan and colleagues constructed a different live adenovirus vectored vaccine that contains the conserved antigenic epitopes of H5N1 influenza virus, including a multi-epitope (ME) consisting of M2 ectodomain (M2e), hemagglutinin (HA) fusion domain (HFD), the T-cell epitope of nucleoprotein (TNP), and HA α-helix domain (HαD) (110). This adenovirus vectored ME vaccine induced humoral and cellular immune responses, reduced the influenza viral titers in the lung of vaccinated mice and showed relatively good immune-protective effects against challenge with H5, H7, and H9 influenza viruses. However, the influenza virus titers in the lung of mice immunized with the Ad-ME vectored vaccine upon challenge were still significantly higher than those of the matched HA control vaccinated animals, suggesting that optimal antibody-mediated protection might require structurally intact HA immunogens. For this reason, other kinds of Ad-based vaccines which express conformationally intact, full length HAs as immunogens can be computationally designed that are based on a concept known as computationally optimized broadly reactive antigen (COBRA) (111–113).

Currently, type 5 AdV (Ad5) is the most commonly used human adenovirus (AdV) vector. Wu and colleagues (114) constructed a replication-defective recombinant AdV (rAd5-avH1HA) that could express the HA protein of the H1N1 influenza virus (A/swine/Zhejiang/199/2013 strain) after a single round of cellular infection. The authors showed that this vaccine induced the production of high-titer neutralizing antibody and significantly reduced replication of the challenged influenza viruses in the nasal cavity and lungs of the vaccinated BALB/c mice. Because most people are naturally immune to Ad5 (115–117), non-human derived recombinant adenovirus vectors, such as bovine adenovirus (BAdV), have also attracted increasing attention among researchers (118). For example, Ekramy and colleagues (119) used BAdV-3 to construct a recombinant adenovirus vectored influenza vaccine BAd-H5HA that expressed the HA gene of H5N1 subtype avian influenza virus and compared the immune effects with the HAdV type C5 (HAdV-C5) vector H5N1 vaccine (HAd-H5HA) in mice. They found for the first time that low dose (1×10 (6) PFU) intramuscular injection of BAd-H5HA could induce strong humoral and cellular immune responses and provide effective immune protection against the heterologous H5N1 virus in mice. In contrast, higher doses (1×10 (8) PFU) were required for HAd-H5HA to achieve comparable immune effects.

### Arenavirus Vectored Influenza Vaccines

Arenavirus is an enveloped RNA virus with a bi-segmented RNA genome (120). Arenaviruses include lymphocytic choriomeningitis virus (LCMV), Lassa virus (LASV), Junin virus (JUNV), Machupo virus (MACV), Pichinde virus (PICV) among others (120, 121). These arenaviruses (also known as mammarenaviruses) can be transmitted to humans through feces, urine, blood, or saliva of infected rodents, which serve as their natural reservoirs. Severe hemorrhagic fever is a major infection-related symptom caused by pathogenic human viruses, such as LASV, JUNV and MACV (122, 123). Contrary, many other arenaviruses, such as PICV, do not induce any clinical manifestations in humans and mammals after infection, and they therefore are considered non-pathogenic (i.e., innocuous) in these accidental hosts (124). Similarly, LCMV generally does not pose serious health threat to the general population as most acquired cases of LCMV infection are non-symptomatic or mild.

Previous studies have demonstrated that arenaviruses mainly infect important immune cells, such as dendritic cells (DCs) and macrophages, at the early stage of infection, which make them an ideal viral vector for vaccine development as they target these antigen-presenting cells (APCs) that can naturally enhance immune responses against the antigenic proteins (125, 126). Several studies have utilized reverse genetics technology to generate recombinant LCMV carrying antigenic proteins of interest (127, 128). Emonet and colleagues (129) developed a three-plasmid reverse genetics system to generate a tri-segmented LCMV. The recombinant LCMV recovered by this system could simultaneously carry up to two transgenes of antigenic protein(s) of interest (126, 129). In June 2018, Hookipa Biotech AG (“Hookipa”), a clinical-stage biotech company specializing in an innovative class of active immunotherapies for oncology and infectious diseases and Gilead Sciences, Inc. (“Gilead”), a research-based biopharmaceutical company, announced a research collaboration and licensing agreement that grants Gilead exclusive rights to Hookipa’s TheraT^®^ and Vaxwave^®^ arenavirus vector-based technologies for two major chronic infectious disease indications, hepatitis B virus (HBV) and human immunodeficiency virus (HIV) (**Figure 3**) (130). It is worth noting that TheraT^®^ vector that is based on the LCMV vector and expressing a non-oncogenic fusion of the human papillomavirus 16 (HPV16) E7 and E6 open reading frame (ORFs) as modeled tumor-associated antigens as well as a similar viral construct that is based on the PICV are currently entering clinical phase 1 testing as a repeated prime-boost immunization strategy for HPV 16-positive head and neck cancer (131). This is a first in human (FIH) Phase I (Dose Escalation)/II (Dose Expansion), multinational, multicenter, open-label study which included 140 participants with HPV 16+ confirmed cancers. While this represents a major step forward in using arenavirus vectors, such as LCMV, as therapeutic tumor vaccines, it is important to note that approximately 5% of human population show evidence of prior exposure to LCMV, and as such, there exists the issue of pre-existing (LCMV) vector immunity. Additionally, while most acquired LCMV infections are asymptomatic or mild, congenital LCMV infection can be quite serious that can result in spontaneous abortion and fetal death, or causes brain dysfunction (132, 133). Recent reports have also suggested that LCMV infection of immunocompromised individuals can also result in some serious medical complications (134–137). In some rare instances, LCMV infection can cause certain forms of neurological disorders, such as aseptic encephalitis, encephalitis, meningoencephalitis, or even microcephaly in healthy individuals, and it therefore must be used with cautions (138, 139).

PICV has a similar viral genome structure as LCMV, but unlike LCMV that is found worldwide because its reservoir is the common house mouse of the genus *Mus musculus* (140, 141). PICV is found in rice rats of the genus *oryzomys albigularis* that is endemic only in the Pichinde valley of Colombia, South America (121, 142). Also, unlike LCMV, PICV is not known to cause disease in humans. Dhanwani and colleagues (143) constructed a three-plasmid reverse genetics system for PICV and used it to develop a PICV vectored influenza vaccine. When the transgenes of antigenic proteins of type A influenza virus (HA and/or NP) were inserted into the recombinant PICV vectored vaccine (rP18tri), these PICV vectored influenza vaccines could effectively induce specific humoral and adaptive immune responses against HA and NP, respectively, in vaccinated mice, and could provide long-lasting, effective protective effects that allowed the vaccinated animals to fend off a lethal mouse-adapted strain of influenza virus (PR8) (144). Furthermore, very low levels of neutralizing antibody (nAb) response were found against PICV. This phenomenon of immune evasion can partly be explained by the fact that the glycoprotein (GPC) on the surface membrane of the Pichinde virion is heavily glycosylated, which allows the virus from being recognized by nAbs (145). Accordingly, PICV vectored vaccines have a major advantage over LCMV vectored vaccines as PICV-based vaccines can overcome the issue of anti-vector immunity during prime-and-boost vaccination (125, 144). While still considered in the early stages of development, PICV vector has proven to be relatively safe, can avoid pre-existing immunity due to the fact that PICV is only naturally found in Colombia, and has shown great potential as a viral vector for developing novel vaccines against influenza and other pathogens.

A recent study has shown that viral vectors based on PICV and LCMV can be engineered to deliver tumor-associated antigens, such as the non-oncogenic fusion protein of the HPV16 E6 and E7 genes, in a heterologous prime and boost regimen can effectively induce tumor-specific CTL (CD8+ T cell) responses, eliminate established solid tumors and protect vaccinated mice against tumor re-challenge. The characteristics of arenavirus family members that can induce strong CTL response and low levels of neutralizing antibodies can help overcome the issue of anti-vector immunity that is commonly observed in other viral vectors, e.g. poxvirus and adenovirus vectors. Based on these exciting new findings, a new arenavirus-based immunotherapeutic strategy has been established (146).

### Newcastle Disease Virus Vectored Influenza Vaccines

Newcastle disease virus (NDV), a single-stranded negative-sense RNA virus, belongs to the *paramyxoviridae* virus family, which encodes six structural proteins (NP, P, M, F, HN, and L) successively from the 3’ end to 5’ end of the viral genome (147). The genome of NDV can be engineered to stably express large segment of the transgenes of interest (of 4.5 kb or larger) and can also express over 3 different non-NDV proteins (102). Theoretically, any foreign genes inserted between any two protein coding sequences of NDV can be expressed. Previous studies have demonstrated that the expression level was highest when the foreign genes are inserted between the NDV P and M genes (148, 149).

NDV can infect respiratory epithelial cells of different mammals, such as poultry, humans, pigs, mice, and non-human primates (NHPs). NDV can activate the local mucosal immunity of the host to induce highly efficient humoral and cellular immune responses (**Figure 3**) (100, 150–152). In addition, the entire replication cycle of NDV is in the cytoplasm, and thus, minimizing the risk of recombination between the viral genome and the host genome (102, 153). NDV vectored vaccine application can be performed by large-scale immunization methods, such as spraying and/or adding the vaccine into drinking water (154). An additional advantage is the high-efficient proliferation of NDV in chicken embryo that favors large-scale production of recombinant NDV vectored vaccines (155). NDV vector has therefore become an important subject for vaccine development for humans and animals (102).

Humans are not the natural hosts of NDV, and NDV therefore is not too pathogenic to humans (154). In addition, it has already been demonstrated that NDV has low amino acid homology and similarity with various known human paramyxoviruses, such as a respiratory syncytial virus (RSV) and human parainfluenza virus (hPIV). Therefore, human immunity against these other respiratory viruses has a limited impact on NDV (153, 156). Bukreyev and colleagues (150) constructed a recombinant NDV to express the HN protein of type 3 human parainfluenza virus (HPIV3), which was then used as the live viral vectored vaccine (at 10^6.5^ PFU) in nasal and intratracheal vaccination of African green monkeys and rhesus monkeys. The live recombinant NDV vectored HPIV3 vaccine only replicated in the respiratory tract of the immunized animals. A second immunization was performed 28 days later. The findings showed that serum titers of neutralization antibodies were significantly higher in animals that received immunization of live recombinant NDV vectored HPIV3 vaccine than immunization with the wide-type HPIV3. To evaluate the effectiveness of NDV vectored vaccines on human influenza virus, Nakaya and colleagues (157) used the NDV Hitchner B1 strain as the viral vector and inserted the HA gene of human influenza A/WSN/33 virus between the NDV P and M genes to construct the rNDV/B1-HA vaccine. The results showed that the recombinant NDV vectored influenza vaccine could induce potent humoral immune responses in vaccinated mice as evidenced by a high level of antibody titers against the HA protein of the influenza virus to provide protective effects against A/WSN/33 virus. DiNapoli and colleagues (158) investigated the immune effects of NDV vectored vaccine against highly pathogenic avian influenza virus (HPAIV) in African green monkeys when they immunized the animals with 10 (7) PFU of a live recombinant NDV vectored vaccine expressing the H5N1 HPAIV HA protein (NDV-HA). High-titer neutralization antibodies were induced after two vaccine inoculations, and high levels of mucosal immunoglobulin IgA responses were also induced in the respiratory tract. These findings demonstrate that NDV vectored influenza vaccines can potentially be promising for human use.

Both Newcastle disease and avian influenza are important infectious viral diseases that seriously endanger the poultry industry. Consequently, preventing both of these diseases through vaccination is of particular interest. Constructing highly efficient bivalent vaccines for Newcastle disease and avian influenza could reduce the costs of vaccine production and adverse stresses of animals by the administration of multiple vaccines (159). NDV vectored AIV vaccine is used as one of the strategies for addressing this issue. Ma and colleagues (160) constructed a recombinant NDV virus expressing A/chicken/Iowa/04-20/2015 (H5N2) virus HA protein for use as an influenza vaccine candidate. The authors found that both live and inactivated NDV vectored influenza vaccines could induce neutralization antibodies against H5N2 HA protein and NDV LaSota strain two weeks after inoculation. However, the immunogenicity of the live NDV vectored influenza vaccine was lower than the inactivated NDV vectored influenza vaccine. In addition, those vaccines showed effectiveness against replication of the highly pathogenic H5N2 A/turkey/Minnesota/9845-4/2015 virus as demonstrated by limited virus shedding from the vaccinated animals (160). The serum antibodies of the immunized chickens could also neutralize highly pathogenic H5N1 and H5N8 viruses after virus challenge. In a separate study, Xu and colleagues (161) fused the ectodomain of the HA protein of H9 subtype AIV with the transmembrane domain and cytoplasmic tail regions of the NDV F protein to construct the recombinant NDV vectored influenza rmNA-H9F vaccine candidate. After immunization, this vaccine protected chickens against a challenge with lethal doses of NDV and AIV H9N2 and induced the production of high titers of NDV-HI, H9- HI, and secretory IgA.

Although NDV vectored AIV vaccines have already been shown to have significant positive immune effects in laboratory experiments, the existence of maternally derived antibodies against NDV could reduce the beneficial immune effects of NDV vectored vaccines (98) (**Figure 3**). To address this issue, several groups of investigators have suggested to use a chimeric NDV vector. A chimeric NDV vector (NDV strain BeaudetteC) has been constructed by replacing the ectodomains of NDV F and HN protein with the correspondence of the avian paramyxovirus serotype 2 (APMV-2) that has different serological characteristics (154). This bypassed the pre-existing maternal antibodies against NDV and provided effective protection against the HPAI virus (162). Chowdhury and colleagues (163) also used this method to construct a recombinant chimeric NDV vector expressing the conserved sequences of the H7 HA protein. Their findings showed that due to maternally derived antibodies’ influence, high titer NDV LaSota specific antibodies were not induced in 4-week-old chicken after heterologous prime and boost vaccination strategy. However, the titer of H7 neutralization antibody was already >16 HI after heterologous prime immunization with NDV/HA, which could protect broiler chickens and turkeys against H7N8 HAPI and also reduced virus shedding.

### Baculovirus Vectored Influenza Vaccines

Baculovirus is a virus that infects insect cells. As such, only invertebrates could be infected by this virus. *Autographa californica* multiple-capsid nuclear polyhedrosis virus (AcMNPV) is the most commonly used baculovirus in research laboratories. AcMNPV is a large double-stranded DNA virus with a genome of 133.9 kb, consisting of 156 open reading frames (ORFs) (164). The genome of AcMVPV is large and thus has sufficient places to insert foreign genes of interest for expression in the virus-infected cells (165, 166). Over the past three decades, baculovirus expression vector systems (BEVS) have been continuously optimized for this purpose. In the last decade, investigations into the use of BEVS-based vaccines have gradually increased with 7 commercialized products have been approved for human use, among which are human papillomavirus vaccine, influenza vaccine, prostate cancer vaccine, and lipoprotein lipase deficiency vaccine (167–169).

With the continuous advancement of BEVS, these techniques have also been applied toward the construction of baculovirus vectored influenza vaccines. Influenza-Flublok Quadrivalent, a quadrivalent, recombinant baculovirus vectored influenza vaccine, formulated to contain 180 μg per 0.5 mL dose, with 45 μg HA of each of the following 4 influenza virus strains: A/Hawaii/70/2019 (H1N1), A/Minnesota/41/2019 (an A/Hong Kong/45/2019-like virus) (H3N2), B/Washington/02/2019 and B/Phuket/3073/2013), is available for human vaccination during the 2020–2021 influenza season (https://www.fda.gov/media/123144/download). Flublok Quadrivalent is a vaccine indicated for active immunization against disease caused by influenza A subtype viruses and type B viruses. It has been approved for use in persons 18 years of age and older in 2017 (170). An earlier trivalent version of Influenza-Flublok was licensed in 2013 but was later replaced by the quadrivalent version. Compared with the inactivated influenza vaccines, the relatively high influenza antigen levels in Flublok formulation could enhance the vaccines’ immunogenicity and reduce the local reactogenicity (171, 172). Additionally, Liu and colleagues (173) evaluated another BEVS-based H5 subunit vaccine (A/goose/Guangdong/1/96) and found that this vaccine could protect BALB/c mice as well as specific pathogen-free (SPF) and commercial broiler chickens, thus suggesting that it could be used as a candidate H5 influenza vaccine strain for humans and animals. Moreover, Yu and colleagues (174) used BEVS to construct a recombinant baculovirus vectored BmNPV-CMV/THB-P10/CTLT vaccine that contains the fused codon-optimized sequence of T lymphocyte epitopes (CTLT) from H1HA, H9HA, and H7HA AIV subtypes, and another fused codon-optimized sequence of Th and B cell epitopes (THB) from H1HA, H9HA, and H7HA AIV subtypes, which was then used to immunize mice and chickens. Their findings demonstrated that the vaccine could activate influenza virus-specific humoral and cellular responses in both species of the animal, suggesting that the vaccine could be used as a universal vaccine against different subtypes of AIV.

Although BEVS has distinct features that make it as an attractive vector platform for vaccine development, when considering BEVS-associated factors such as protein complexity, post-translational modification, scale and cost, it is not ideally suited for all vaccine product developments (168, 175). The main limitation in applying baculovirus vector vaccine as a commonly used vaccine vector platform is the lack of safety guidelines for human immunization. Further study is required to successfully integrate the technology into real-life situations. It is also necessary to establish the complete baculovirus gene expression in a mammalian system in order to further the safety profile of baculovirus-based vaccines. Baculovirus might be the next-generation vaccine for influenza with some of their attractive attributes as a potential vaccine (176).

### Herpesvirus Vectored Influenza Vaccines

Herpesvirus is a virus with a large double-stranded DNA genome that ranges from about 125 to 240 kbp (177). These herpesviruses are restricted in host range, e.g., avian herpesviruses can only infect avian species (**Figure 3**). Several herpesvirus vectors are available, including the herpesvirus of turkeys (HVT), avian infectious laryngotracheitis virus (ILTV), and Marek’s disease virus (MDV) of chicken. MDV vaccine has been used in poultry industries for over 40 years as it can induce life-long immunity against Marek’s disease (178). HVT has been used as a virus vector to express various antigenic proteins, such as HA protein of AIV and F protein of NDV (179, 180). Several HVT vector-based commercial vaccines expressing the HA protein of H5 subtype AIV, such as Vectormune^®^ AI [CEVA Animal Health, Lenexa, KS, USA (www.vectormune-ai.com)], have been developed. This vaccine provides effective immune protections for turkeys and domestic ducks against H5 subtype HPAI (181, 182). Kapczynski and colleagues (182) constructed a recombinant HVT vectored AIV vaccine expressing the clade 2.2 H5N1 AIV HA protein, which induced significant protective effect in chickens after H5N1 and H5N2 HPAIV challenges that could achieve the survival rate of the animals by 80%-95%. In addition, splenic T lymphocytes from HVT-vector-AI vaccinated chickens recognized MHC-matched target cells infected with H5, H6, H7, or H9 AI virus. Li and colleagues (179) constructed another recombinant HVT vectored vaccine expressing H7N1 HPAIV HA gene (rHVT-H7HA) by using the bacterial artificial chromosome (BAC) cloning technique (82). Immunization with rHVT-H7HA induced the production of H7-specific immune responses to help chickens fend off the infection of highly pathogenic H7N1 virus and significantly reduced virus shedding. Liu and colleagues (183) also used the BAC cloning technique and CRISPR/Cas9 gene-editing technique to construct a recombinant HVT vectored vaccine expressing H9 HA protein. Immunization with rHVT-H9 could induce humoral and cellular immune response in chickens after 35 days of vaccination as evidenced by the high titer of neutralization antibody HI against H9, and activated IFN-γ+ CD4+ and IFN-γ+ CD8+ T cells. When the vaccinated animals were challenged with the H9N2 AIV at 35 days post vaccination, no virus shedding in the tracheal and cloacal swabs of chickens was observed at 3 and 5 days post challenged. In addition, no H9N2 virus was detected in the organs (trachea, lung, spleen, and kidney) of the vaccinated chickens. These findings demonstrated that HVT has promising prospects for use as a viral vector for avian influenza vaccine developments. However, some disadvantages of herpesvirus vector, such as high level of pre-existing anti-vector immunity (184, 185), requiring relative longer time to provide full protection by *in ovo* route (186) and the potential risk caused by lifelong infections in hosts cells can be an impediment when considering this viral vector in vaccine developments (187).

## Development of Seasonal Influenza Vaccines and Universal Influenza Vaccines

Many of the aforementioned viral vectored vaccines have proven to be safe and immunogenic, which can activate both humoral and cellular immune responses against the antigenic proteins of interests at either the local mucosal sites or systematically. Thus, virus vectored vaccines can overcome some of the inherent disadvantages of traditional inactivated vaccines, attenuated vaccines, and subunit vaccines. However, the long-term effectiveness and safety of some of these vaccines still need to be further investigated. A real challenge in influenza vaccine development is how to tackle the frequent antigenic drift and antigenic shift, and the rapid mutation rates of influenza viruses. In order to overcome this problem, recent efforts have focused on improving the forecast of circulating influenza viruses in order to develop vaccine candidates that can closely match those forecast seasonal viruses, and on developing universal influenza vaccines that can provide broadly protective efficacy against multiple strains of the influenza virus.

### Development of Seasonal Influenza Vaccines

Influenza surveillance is an annual public health activity done around the world. This coordinated annual global effort to track influenza virus infections had been organized by the World Health Organization (WHO) called the Global Influenza Surveillance Network (GISN) in 1952, which was renamed as the Global Influenza Surveillance and Response System (GISRS) in 2011 and expanded to include 143 institutions in 113 Member States (188). According to the main two approaches of this influenza virus surveillance system, information from outpatient clinics or hospitals was collected through syndromic surveillance systems to monitor the emerging occurrence of influenza illnesses at the local community, while virologic characteristics of circulating influenza virus strains were collected by the laboratory surveillance systems (189). Twice a year, all of the collected information on global seasonal influenza virus circulations are assimilated, analyzed by the GISRS, and provided to the appropriate authorities for the seasonal influenza vaccine strain selection and for universal influenza vaccine development. In the past decades since 1950s, the influenza virus vaccine strain selection relied on the data of annual serological assays which provide a phenotypic measurement of how immune system reacts to the contemporary circulating influenza viruses. However, serological assays have certain disadvantages that include labor intensiveness, inconsistent data across labs, and data that are not publicly available and/or are difficult to interpret or scale up. These methods therefore can only be applied to analyze a small subset of influenza viruses. As such, it is imperative to develop tools that can analyze the ongoing evolution of influenza viruses that is based on the surveillance data and to provide recommendations for selecting influenza vaccine strains with potentially greater and broader protection to the general public. Phylogenetic tree or evolutionary tree is one of the tools used to visualize the genetic evolution of circulating influenza viruses. Over the years, researchers have developed multiple similar tools to generate phylogenetic trees and analyze the genetic evolution of circulating influenza viruses. One such method is called Nextflu (https://bedford.io/projects/nextflu/), an online visualization tool that was designed by Neher and Bedford (190). Nextflu can perform near real-time tracking of circulating influenza virus evolution, especially on seasonal influenza virus evolution in humans. The users can analyze seasonal influenza circulation patterns by visualizing the genetic and epidemiological features of the circulating influenza virus strains, which can provide projections for the upcoming flu season. Similarly, the AD plot of a population-level influenza viral sequence combines information from phylogenetic inference and ancestral character state reconstruction (191), a method that relies on mapping the character states of living organisms onto phylogenies using the method of maximum parsimony in order to identify alleles and associated genetic changes that might be under directional selection (190). Besides phylogenetic tree, antigenic cartography or antigenic maps are also important analysis tools. Antigenic cartography has been developed using data on human influenza virus subtype A (H3N2) and is routinely used to analyze the global data from the WHO influenza surveillance network as part of the influenza vaccine-strain selection process (192). Antigenic maps are based on serological data that reflect the antigenic properties of the pathogens (193). Antigenic characterization of influenza viruses is typically based on hemagglutination inhibition (HI) data for viral isolates tested against strain-specific post-infection ferret antisera (194). By using the aforementioned tools to visualize the natural evolution of influenza viruses, researchers can build statistical learning models to explain antigenic difference and to predict the next evolutionary step of the influenza virus (195). For example, Steinbrück and colleagues combined AD plots and antigenic tree to analyze the evolution of HA genes of influenza A (H3N2) collected from 2002 to 2007, and successfully predicted the antigenic evolution of the H3N2 viruses in retrospective testing scenarios (196).

Despite the ability to forecast the next seasonal influenza virus strains, a period of about one year is typically needed in order to complete the selection of the influenza vaccine strains and to bring them through vaccine production, release, and distribution processes. During this period of time, however, the circulating influenza virus might further mutate to render it dissimilar from the selected vaccine strain as demonstrated by recent examples with the predicted influenza vaccines for the H3N2 virus (197), which evolved particularly fast and unpredictably compared to other seasonal influenza viruses and rendered the seasonal influenza vaccines unmatched with the naturally circulating H3N2 strains in six of the past fifteen influenza seasons from 2004 to 2019 (197). Additionally, the antibody level stimulated by the seasonal influenza vaccine may be maintained only for a relatively short period of time before it reaches a sub-protective level prior to the end of the flu season (198). These issues can complicate the power of prediction and production of an optimal seasonal influenza vaccine and therefore might necessitate other methods (e.g., universal influenza vaccine development) to produce an influenza vaccine with broader coverage for different strains of the influenza virus.

### Development of Universal Influenza Vaccines

**Figure 4:**
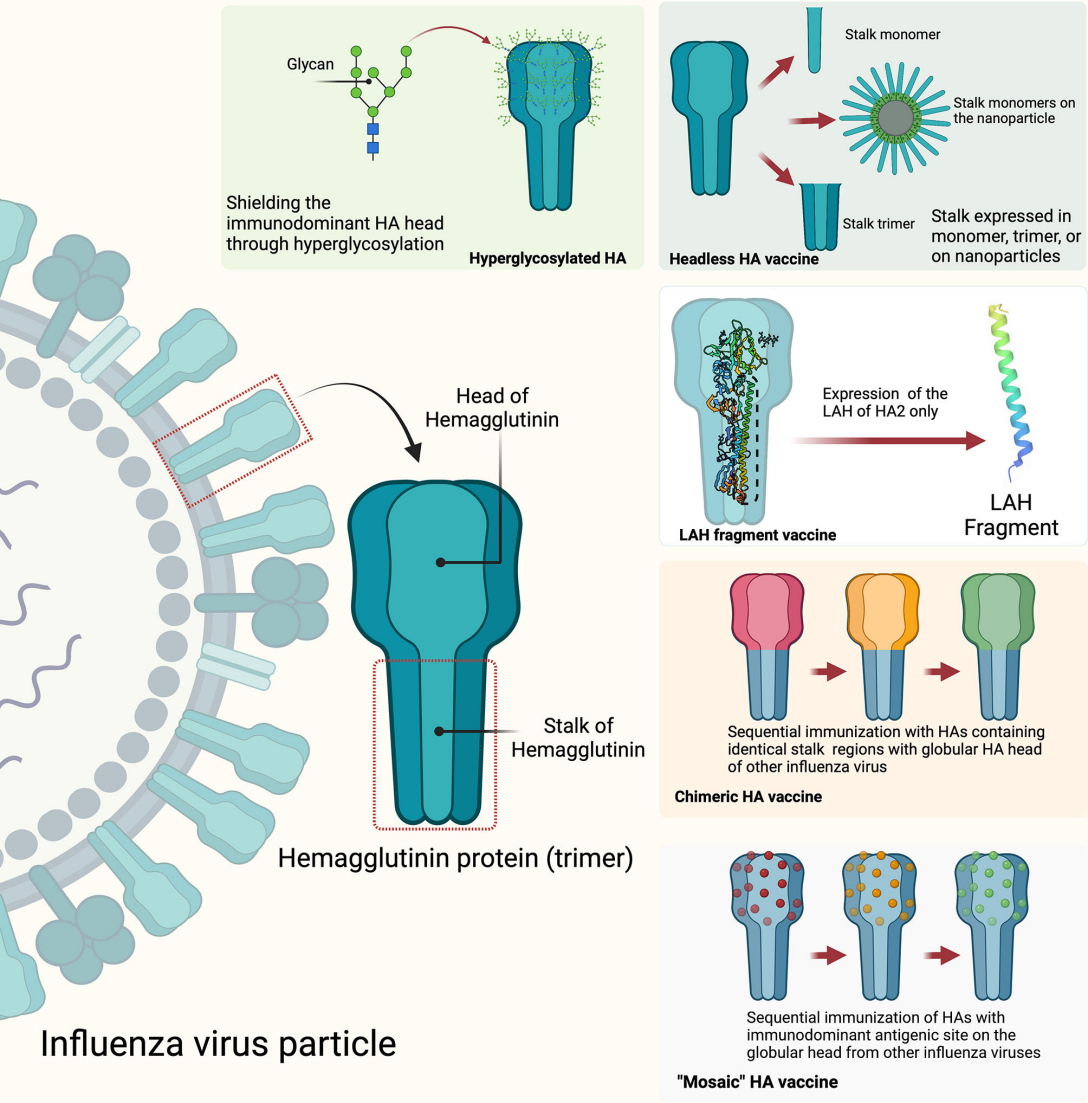
Strategies to develop HA stalk-directed universal influenza vaccines: hyperglycosylated HA, headless HA, long alpha helix (LAF) fragment, chimeric HA, and mosaic HA. The figure was created with Biorender.com.

Universal influenza virus vaccines are vaccines that are meant to provide protective coverage against influenza A and influenza B viruses irrespective of their known propensity for antigenic drift and shift of the HA, NA, or other viral genes or gene segments. Currently, several common targets for universal influenza vaccine developments include the conserved stalk domain of the HA protein, the ectodomain of the M2 ion channel (M2e) and the internal proteins nucleoprotein (NP) and matrix protein (M1) (199, 200). Since the stalk region of the influenza virus is located on the surface of the virion in a region that is not readily assessable to neutralizing antibodies, it is therefore facing less selective pressure from the host immune system and therefore is highly conserved among influenza viruses. It is therefore reasonable to assume that targeting this conserved stalk region of HA protein could induce broadly reactive antibodies against influenza viruses within subtypes and between multiple subtypes within a group and even between groups (201). Different strategies have been explored that include generating candidate universal influenza vaccines with hyperglycosylation of the HA head, “headless” HA, expression of just the long alpha helix (LAH) domain of the stalk, and development of chimeric and mosaic HAs to induce stalk-directed antibodies (**Figure 4**) (200). Some of the universal influenza vaccines developed through some of these strategies have entered into human clinical trials and showed some encouraging results. For example, results of a phase I clinical trial that included 65 participants who received two immunizations with different combinations of chimeric HA live-attenuated influenza virus, inactivated influenza virus, or inactivated influenza virus plus adjuvant have shown evidence of induction of anti-stalk H1 antibodies. More encouragingly, the results showed that the induced anti-stalk antibodies persisted up to 4-fold above baseline for 1.5 year in those participants who received two doses of chimeric HA inactivated and adjuvanted influenza virus vaccines (202, 203).

Other attempts to produce a universal influenza vaccine have involved the influenza viral M2e protein as the target. While the specific neutralizing antibody induced by the influenza viral M2e protein can bind to M2e expressed on virus-infected host cells through complement or antibody-dependent cell-mediated cytotoxicity, some studies have shown that M2e-specific serum antibody titers decline rapidly in the vaccinated individuals (204). Such an example of weak antigen immunogenicity coupled with the fact that most of the induced antibodies are non-neutralizing antibodies highlight some of the challenges of a universal influenza-vaccine development process. It has therefore been suggested that by studying the more conserved influenza viral antigens (or the conserved regions of certain viral antigen) in combination with the use of appropriate adjuvants and other methods to enhance antigen immunogenicity (205), and/or by studying the combined use of universal influenza vaccines with other known vaccines, these methods might help enhance the potential for the production of more durable and broadly protective HA/NA neutralizing antibodies against different strains of the influenza virus (206).

## Recombinant Influenza Viruses as Viral Vectors for Vaccine Developments

Reverse genetics systems are available for generating recombinant influenza viruses in the laboratory (207, 208), and are the basis for use as a vaccine vector. IAV infection can induce CD4+ and CD8+ T cell responses to produce cytokines (209, 210). Certain cytokines can then mediate CD4+ T cells to differentiate into T helper 1 cells (Th1) or T helper 2 cells (Th2) (211, 212). Th1 cells participate in CD8+ cytotoxic T cell-mediated immune responses, recognize and disrupt virus-infected cells, and inhibit virus replication and proliferation (211, 212). On the other hand, Th2 cells can activate B cells to promote the production of neutralization antibodies, thus participating in the host’s humoral immunity (213, 214). Live attenuated influenza vaccines (LAIV) can also induce mucosal immunity in the early stage of infection (215). In recent years, recombinant influenza viruses have been used as virus vectors.

Different cloning strategies of the foreign genes into the influenza virus genome could result in different immune responses. For example, some studies have demonstrated that when the foreign genes are inserted into the gene segments of the influenza viral HA or NA, the recombinant influenza virus vectored vaccine could mediate strong neutralization antibody responses against the foreign proteins. In contrast, inserting foreign genes into the gene segments of the influenza viral internal proteins (e.g., NP and M1) or gene segments encoding ribonucleoproteins (e.g., PB2, PB1, and PA) could induce CD8+ T cell responses against the foreign proteins (216). Based on these findings, Isakova-Sivak and colleagues (217) constructed two recombinant live influenza virus vectored AdV vaccine (LAIV-AdV) by inserting the T-cell epitopes of AdV into the ORF of the full-length NA gene and truncated NS1 gene of the licensed cold-adapted H7N9 LAIV virus. Immunization with this LAIV-AdV vaccine induced relatively strong influenza virus-specific antibodies and cell-mediated immunity in C57BL/6J mice, which could sufficiently protect the vaccinated animals from virulent H7N9 influenza virus and human Adv5 infection. The target cells loaded either with influenza NP366 or AdV DBP418 peptides were recognized and efficiently killed by the CD8 T-cell responses induced by both LAIV-AdV candidates. In addition, high levels of memory T cells which targeted to the immunodominant H2b-restricted CD8 T-cell epitope were detected in the immunized mice after the AdV5 challenge. Furthermore, Matyushenko and colleagues (218) constructed two recombinant LAIV vectored vaccines by inserting the genes encoding T cell epitopes of RSV M2 protein into the neuraminidase (NA) or non-structural protein (NS1) of H7N9 subtype influenza virus. Both of the recombinant LAIV vectored vaccines could simultaneously protect mice from infection by influenza virus and RSV and activated influenza- and RSV-specific CD4+ and CD8+ T cell responses in the lung and lung tissue-resident memory T cell (TRM) subsets of the vaccinated and challenged animals. Despite these positive results, the safety and effectiveness of recombinant influenza virus vectored vaccines still need further investigations and improvements. As a live attenuated influenza virus, it can still replicate when used as a virus vector, and as a result, there is still the possibility of gene recombination and reassortment with the circulating wild-type influenza virus (53). In addition, the foreign genes could be lost when the virus vectored vaccine are continuously passaged in the laboratory or in vaccinated individuals (219).

## Closing Remarks

Many of the viral vectored influenza vaccines have proven to be safe and immunogenic, which can activate both humoral and cellular immune responses against the antigenic proteins of interests at either the local mucosal sites or systematically. These virus vectored influenza vaccines can therefore overcome some of the inherent disadvantages of traditional influenza subunit vaccines, inactivated vaccines, and attenuated vaccines. So far, many virus vectored influenza vaccines have been investigated, with some showing acceptable levels of effectiveness. These novel viral platforms and technologies provide rapid, highly efficient, and cost-effective means to produce vaccines for many diseases of humans, livestock as well as companion and exotic animals (220). However, the long-term effectiveness and safety of some of these vaccines still need to be further investigated. A real challenge in influenza vaccine development is how to tackle the frequent antigenic drift and antigenic shift, and the rapid mutation rates of these viruses. A potential solution to this vexing but essential issue is through the process of optimizing the methods of predicting the next major strain of the influenza virus so that a better seasonal vaccine can be developed with maximal protective value and/or through the development of universal influenza vaccines, which are still under intense investigative and developmental stages.

## Author Contributions

Writing and original draft preparation, JC and JW. Writing, reviewing, and editing, HL and JZ. Figures preparation, JC. Funding acquisition, JC and JZ. All authors contributed to the article and approved the submitted version.

## Funding

This study was supported by the National Natural Science Foundation of China (NSFC) (grant number 31800141), the Natural Science Foundation of Guangdong Province, China (grant number 2017A030310573), the Rural Revitalization Strategy Special Foundation of Guangdong Province (Rural Science and Technology Commissioner Program) (grant number 163-2019-XMZC-0009-02-0075), and Key Research Program of Education Department of Guangdong Province (grant number 2020ZDZX1018).

## Conflict of Interest

The authors declare that the research was conducted in the absence of any commercial or financial relationships that could be construed as a potential conflict of interest.
